# Early Pep-13-induced immune responses are SERK3A/B-dependent in potato

**DOI:** 10.1038/s41598-019-54944-y

**Published:** 2019-12-05

**Authors:** Linda Nietzschmann, Karin Gorzolka, Ulrike Smolka, Andreas Matern, Lennart Eschen-Lippold, Dierk Scheel, Sabine Rosahl

**Affiliations:** Department Biochemistry of Plant Interactions, Leibniz Institute of Plant Biochemistry, Weinberg 3, D-06120 Halle (Saale), Germany

**Keywords:** Plant immunity, Secondary metabolism

## Abstract

Potato plants treated with the pathogen-associated molecular pattern Pep-13 mount salicylic acid- and jasmonic acid-dependent defense responses, leading to enhanced resistance against *Phytophthora infestans*, the causal agent of late blight disease. Recognition of Pep-13 is assumed to occur by binding to a yet unknown plasma membrane-localized receptor kinase. The potato genes annotated to encode the co-receptor BAK1, *StSERK3A* and *StSERK3B*, are activated in response to Pep-13 treatment. Transgenic RNAi-potato plants with reduced expression of both SERK3A and SERK3B were generated. In response to Pep-13 treatment, the formation of reactive oxygen species and MAP kinase activation, observed in wild type plants, is highly reduced in *StSERK3A/B*-RNAi plants, suggesting that StSERK3A/B are required for perception of Pep-13 in potato. In contrast, defense gene expression is induced by Pep-13 in both control and StSERK3A/B-depleted plants. Altered morphology of *StSERK3A/B*-RNAi plants correlates with major shifts in metabolism, as determined by untargeted metabolite profiling. Enhanced levels of hydroxycinnamic acid amides, typical phytoalexins of potato, in *StSERK3A/B-*RNAi plants are accompanied by significantly decreased levels of flavonoids and steroidal glycoalkaloids. Thus, altered metabolism in *StSERK3A/B-*RNAi plants correlates with the ability of StSERK3A/B-depleted plants to mount defense, despite highly decreased early immune responses.

## Introduction

Perception of pathogen or microbe-associated molecular patterns (PAMPs/MAMPs) in plants by plasma membrane pattern recognition receptors (PRRs) initiates the activation of immune responses, leading to the formation of reactive oxygen species (ROS), MAP kinase activation and transcriptional reprogramming^1^. PRRs have distinct ectodomains; those PRRs recognizing peptides belong to the class of leucine-rich repeat receptor-like kinases (RLKs) or proteins (RLPs) and require the co-receptor BRASSINOSTEROID-INSENSITIVE 1- ASSOCIATED RECEPTOR KINASE 1 (BAK1) or the adapter SUPPRESSOR OF BIR1-1 (SOBIR1^2^), with which they heterodimerize upon ligand binding. BAK1 belongs to the class of somatic embryogenesis receptor kinases (SERKs), which are considered to be integration nodes of different signaling pathways, due to their importance for the perception of exogenous as well as endogenous cues^3^. Thus, in addition to PRRs, SERK3/BAK1 associates with the brassinosteroid (BR) receptor BRASSINOSTEROID INSENSTIVE 1 (BRI1) to mediate BR signaling^4,5^.

In contrast to Arabidopsis, which has five members of the SERK gene family, only three members, SERK1, SERK3A and SERK3B, were reported for tomato (*Solanum lycopersicum*)^6,7^. While both SERK3A or SERK3B are important for defense against root knot nematodes and nonpathogenic *Pseudomonas syringae* pv. *tomato* (*Pst*) DC3000 *hrcC*, a role for defense against pathogenic *Pst* DC3000 was demonstrated by virus-induced gene silencing for SERK3B, but not SERK3A^7^. Moreover, silencing of the *Nicotiana benthamiana* homologs, *Nb*SERK3A and *Nb*SERK3B, resulted in enhanced susceptibility of *N. benthamiana* upon infection with *Phytophthora infestans*, the causal agent of potato late blight, but not with the non-adapted pathogen *Phytophthora mirabilis*^8^. A role for BAK1 in peptide-mediated signaling was shown for recognition of *Phytophthora* elicitins. The receptor-like protein ELICITIN RESPONSE (ELR) from a wild potato associates with SERK3A from *Solanum tuberosum* and confers elicitin recognition and enhanced resistance against *P. infestans*^9^.

Late blight, caused by the oomycete *P. infestans*, is economically the most important disease of potato. As a PAMP of *Phytophthora* species, the oligopeptide Pep-13 elicits defense responses, first characterized in parsley^10^ and subsequently in potato^11,12^. Infiltration of Pep-13 into potato leaves leads to the accumulation of salicylic acid, jasmonic acid and hydrogen peroxide, as well as to defense gene activation, hypersensitive cell death and enhanced resistance to *P. infestans* infection^13^. In parsley, biochemical analyses revealed that Pep-13 is recognized by a plasma membrane-bound receptor^10^. The specificity of eliciting defense responses by variants of Pep-13 is similar in parsley and potato, suggesting a similar mechanism of perception^10,12,13^.

The potato homologue of BAK1 was identified as a Pep-13-activated gene in microarray experiments. Transgenic plants with reduced expression of *StSERK3A/B* displayed altered morphology that was reminiscent of a brassinosteroid-deficiency phenotype and which correlated with differential accumulation of phenolics, flavonoids and sterols in untreated plants. Importantly, *StSERK3A/B*-RNAi plants were unable to activate early defense responses in response to Pep-13 treatment. Despite this, defense gene expression was induced by Pep-13 in *StSERK3A/B*-depleted plants.

## Results

### *StSERK3B* transcript levels are increased by Pep-13 treatment

**Figure 1:**
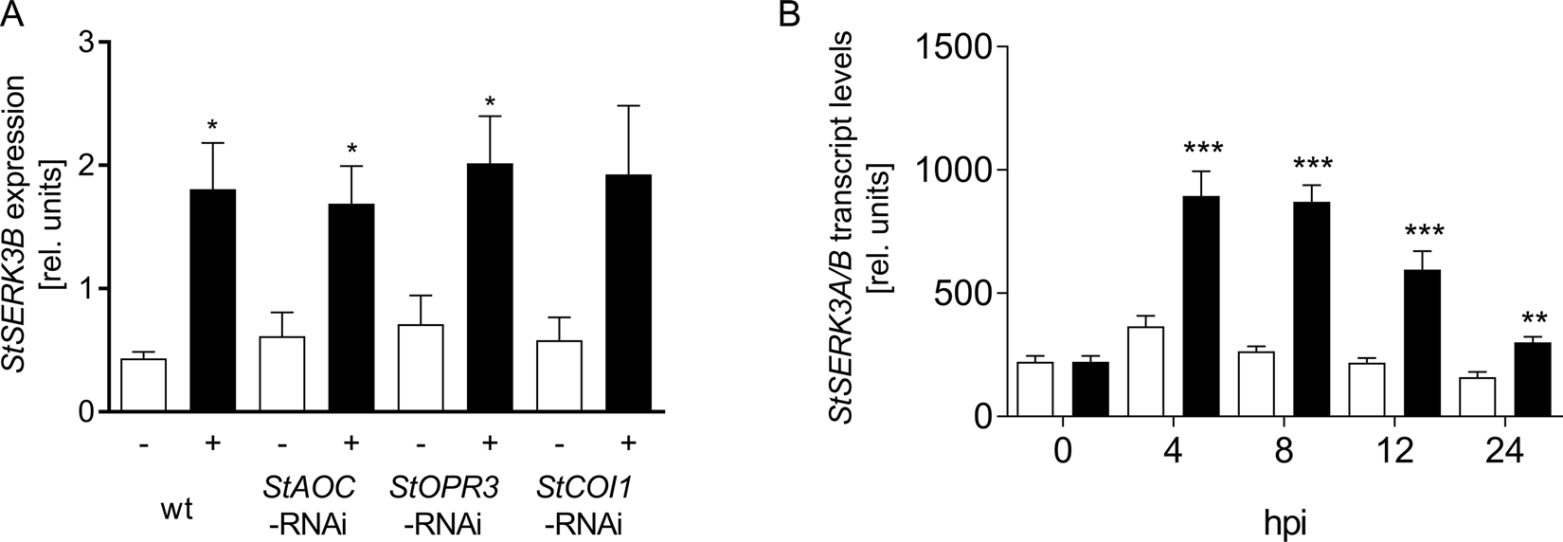
Pep-13-induced *StSERK3A/B* expression. (**A**) Pep-13-induced accumulation of *StSERK3B* transcripts in microarray analyses. RNA from wild type (wt) and transgenic plants with reduced expression of the jasmonic acid biosynthetic genes *StAOC* (*StAOC*-RNAi) and *StOPR3* (*StOPR3*-RNAi) as well as the JA receptor *StCOI1* (*StCOI1*-RNAi), infiltrated with 100 µM W2A (white bars) or Pep-13 (black bars), was used in microarray analyses. Data presented are derived from three independent experiments (n = 3). Statistical analysis was performed using Mann-Whitney two-tailed U test (W2A versus Pep-13-treatment); *p < 0.05. (**B**) Kinetics of Pep-13-induced *StSERK3A/B* expression. qRT-PCR was performed with RNA isolated from leaf disks from wild type potato plants infiltrated with 100 µM W2A (white bars) or Pep-13 (black bars) at the time points indicated. Data are derived from three independent experiments (n = 6). Statistical analysis of Pep-13-induced expression versus W2A treatment was performed using Mann-Whitney two-tailed U test (W2A versus Pep-13-treatment); **p < 0.01, ***p < 0.001.

*StSERK3B* was identified in microarray analyses^14,15^ as a gene activated in response to treatment by Pep-13 in wild type, as well as in transgenic plants impaired in jasmonic acid biosynthesis (*StAOC*-RNAi and *StOPR3*-RNA) or perception (*StCOI1*-RNAi; Fig. 1A). The originally identified EST (MICRO.11825.C1) corresponds to PGSC0003DMT400032797, annotated to encode the receptor kinase SERK3B (Sotub01g042020; http://solanaceae.plantbiology.msu.edu/). The 60mer located on the potato chips^15^, corresponds to the 3′ untranslated region of *StSERK3B*, but not *StSERK3A* (Sotub10g013940). In subsequent qRT-PCR analyses, primers were used, which are predicted to amplify both *StSERK3A* and *StSERK3B* transcripts. These analyses revealed significantly enhanced *StSERK3A/B* transcript levels in Pep-13-infiltrated potato leaves four hours after treatment, which declined after 24 hours (Fig. 1B).

The protein coding regions of *StSERK3A* and *StSERK3B* are located on 11 exons on chromosome 10 and 1, respectively (Supplementary Fig. S1A,B). The full length proteins StSERK3A (KJ625629, 615 amino acids) and StSERK3B (XP_006351807, 617 amino acids) display 79% sequence identity to *At*BAK1 (At4G33430). Protein domain prediction programs describe a similar structure of StSERK3A and B to tomato SERK3B^7^, with a signal peptide, a leucine zipper region, four LRR domains, a proline-rich domain preceding a transmembrane domain and a C-terminal kinase domain (Supplementary Fig. S1C,D). *St*SERK3A and B share 89% and 87% sequence identity at the amino acid (Supplementary Fig. S1E) and nucleotide level, respectively.

### Defense responses in *StSERK3A/B-*RNAi plants

**Figure 2:**
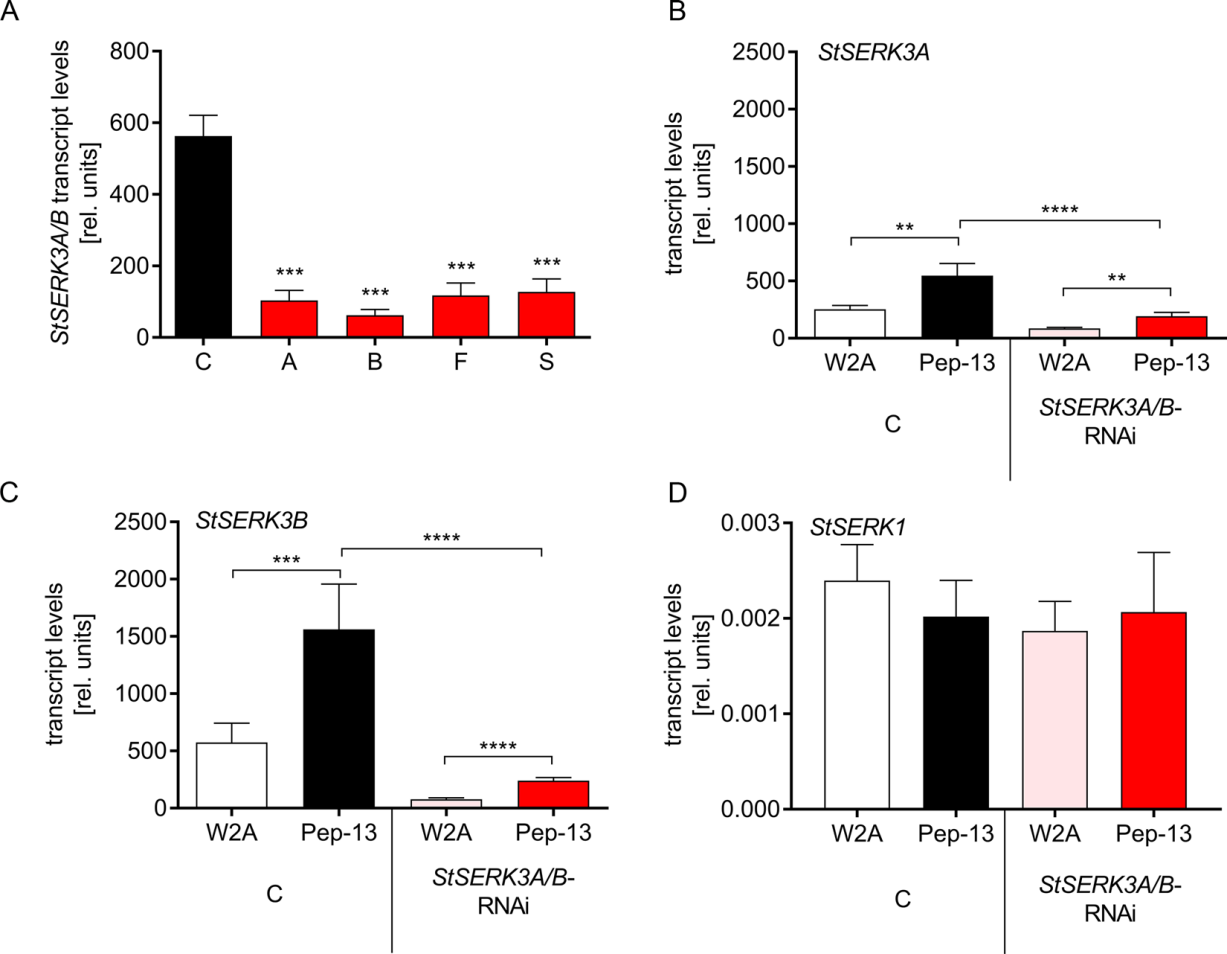
Reduced *StSERK3A/B* expression in *StSERK3A/B-*RNAi plants. (**A**) qRT-PCR was performed with RNA isolated from leaf disks from control (wt and ev) and transgenic *StSERK3A/B-*RNAi plants (A,B,F,S) after incubation in 5 nM Pep-13 for 4 hours. Expression of EF1α was used as a reference. Data are derived from three independent experiments (wt, ev, A,B,F,S: n = 6 each). Statistical analysis was performed using Mann-Whitney two-tailed U test (C versus RNAi plant). ***P < 0.001. (**B**–**D**) qRT-PCR was performed with the same RNA as in (**A**) using specific primers for *StSERK3A* (**B**), *StSERK3B* (**C**) or *StSERK1* (**D**). Statistical analyses was performed using Mann-Whitney two-tailed U test. **P<0.01, ***P<0.001, ****P < 0.0001.

To assess the function of *StSERK3A/B* for Pep-13-induced defense repsonses, RNA interference constructs were generated targeting the 3′ end of the gene (Supplementary Fig. S1B). Due to the high sequence similarity of *StSERK3B to StSERK3A*, the RNAi fragment is predicted to affect the expression of both genes. Transgenic potato plants expressing the RNAi construct were generated by *Agrobacterium*-mediated leaf disk transformation. qRT-PCR was performed with RNA from Pep-13-treated leaf disks of four independent transformants using primers that amplify both *StSERK3A* and *StSERK3B* transcripts. Significantly reduced levels of *StSERK3A/B* transcripts were detected in all plant lines (Fig. 2A). To differentiate between *StSERK3A* and *StSERK3B* expression, gene-specific primers were used. These experiments revealed that, in wild type plants, *StSERK3A* is activated twofold in response to Pep-13, but generally expressed at lower levels than *StSERK3B*, whose transcripts increase threefold (Fig. 2B,C). Importantly, both genes were affected by the RNAi construct, since transcript levels after Pep-13 treatment were significantly lower in the RNAi compared to control plants. Despite this decrease, Pep-13-induced StSERK3A/B transcript levels in the RNAi plants were higher than those induced by W2A treatment (Fig. 2B,C), suggesting that residual levels of StSERK3A/B were sufficient to induce a weak Pep-13-specific response.

Since the RNAi fragment also showed similarity to *StSERK1* (Sotub04g027320), *StSERK1* transcript levels were determined in wild type, empty vector and *StSERK3A/B*-RNAi plants using gene-specific primers. *StSERK1* transcripts did not accumulate in response to Pep-13 infiltration, nor did they show differences between control and *StSERK3A/B*-RNAi plants (Fig. 2D), suggesting that the RNAi fragment specifically reduced the levels of *StSERK3A/B* transcripts.

**Figure 3:**
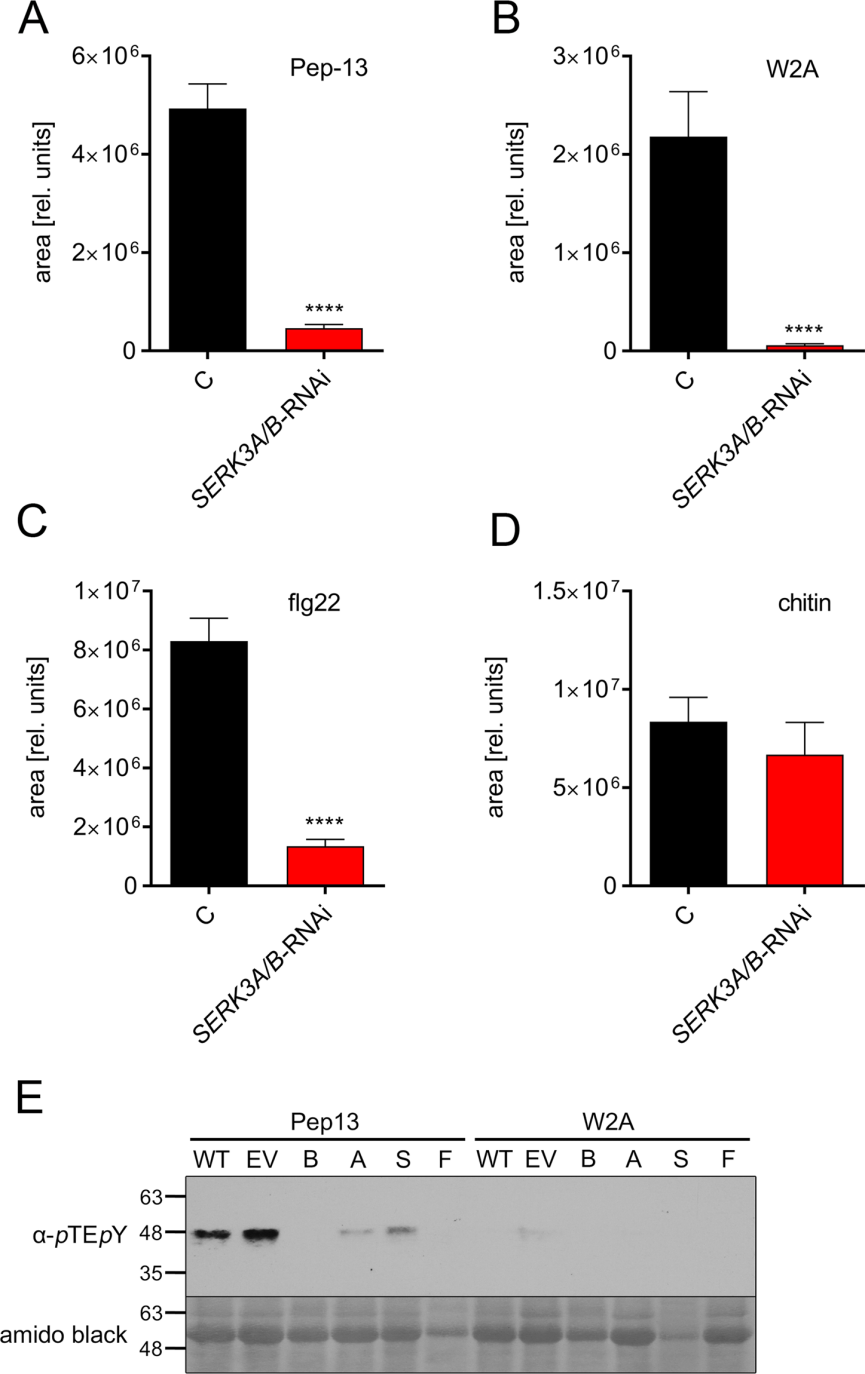
*StSERK3A/B* are required for early defense responses. Leaf disks from control (WT and EV) and *StSERK3A/B-*RNAi plants (A,B,F,S) were incubated in 5 nM Pep-13 (**A**), 5 nM W2A (**B**), 100 nM flg22 (**C**) or 100 µg/ml chitin (**D**) and assayed for luminol-based ROS production. Data show the area under the curve of ROS production (relative units) and are derived from two independent experiments (n ≥ 15). Statistical analyses were performed using two-tailed Mann-Whitney U test (****p < 0.0001). (**E**) Leaves from wild type, empty vector and *StSERK3A/B-*RNAi plants were infiltrated with Pep-13 or W2A and assayed for MAP kinase activation after 10 min. Protein extracts were subjected to Western blot analyses using anti-pTEpY antibodies (upper panel). The membrane was subsequently stained with amido black (lower panel). The experiment shown is representative for two independent experiments.

The formation of reactive oxygen species (ROS), the oxidative burst, is a hallmark of early defense responses. In a luminol-based assay, Pep-13 elicited the oxidative burst in wild type and empty vector plants, but not in *StSERK3A/B-*RNAi plants (Fig. 3A, Supplementary Fig. S2A). Application of the nearly inactive analog W2A did not lead to a strong ROS production (Fig. 3B, Supplementary Fig. S2B). The peptide elicitor flg22, whose activity is BAK1-dependent in Arabidopsis^16,17^, elicited a strong ROS burst in control, but not in *StSERK3A/B-*RNAi plants (Fig. 3C, Supplementary Fig. S2C), suggesting a requirement of *StSERK3A/B* for both PAMPs, Pep-13 and flg22. In contrast, the oligosaccharide chitin, a fungal PAMP, induced ROS formation in a *StSERK3A/B*-independent manner in all plants tested (Fig. 3D, Supplementary Fig. S2D).

The activation of defense-related MAP kinases was monitored by Western blot using an antibody specific for phosphorylated MAPK-pTEpY motifs. Pep-13, but not W2A, induced the activation of a MAP kinase of about 48 kD in wild type and empty vector plants (Fig. 3E, Supplementary Fig. S2E). Importantly, MAPK activation was highly reduced in all *StSERK3A/B*-RNAi lines tested, indicating that *St*SERK3A/B are required also for this early defense response.

**Figure 4:**
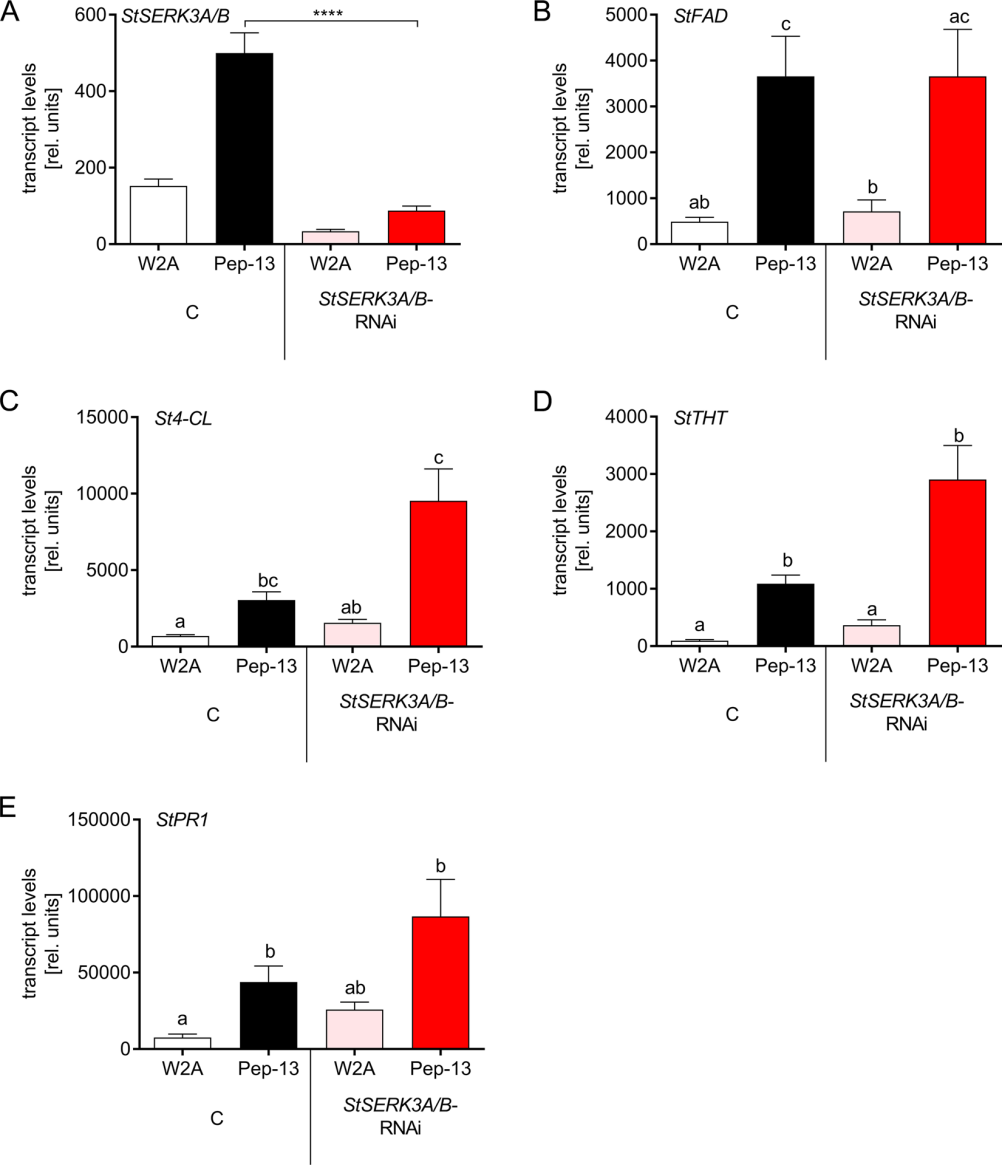
Pep-13 induces defense responses in *StSERK3A/B-*RNAi plants. RNA was isolated from leaf disks from wild type potato plants (white and black bars) or *StSERK3A/B-*RNAi plants (red bars) four hours (24 hours for *StPR1*) after infiltration of 100 µM W2A (open bars) or Pep-13 (filled bars). qRT-PCR was performed with primers sepcific for *StSERK3A/B* (**A**), *StFAD* (**B**), *St4-CL* (**C**), *StTHT* (**D**) and *StPR1* (**E**). Data are derived from three independent experiments (n = 16 for control, n = 32 for *StSERK3A/B-*RNAi plants, except for *StPR1* and *St4-CL* expression: two experiments, n = 8 for control, n = 16 for *StSERK3A/B-*RNAi plants). Statistical analysis was performed using Mann-Whitney two-tailed U test for Pep-13-treated samples (control versus *StSERK3A/B-*RNAi plants) for (**A**) and one way Anova for (**B**–**E**). ****p < 0.0001.

Despite the inability to mount an oxidative burst and to activate MAP kinases, enhanced levels of transcripts of selected defense genes were detected in Pep-13-treated leaf disks. While Pep-13-induced expression of *StSERK3A/B* was highly reduced in *StSERK3A/B-*RNAi plants (Fig. 4A), transcript levels of *FATTY ACID DESATURASE* (*FAD*), *4-COUMARATE-COA LIGASE* (*4-CL*), *TYRAMINE HYDROXYCINNAMOYL-TRANSFERASE* (*THT*) and *PATHOGENESIS-RELATED 1* (*PR1*) were similarly elevated in Pep-13-infiltrated control and *StSERK3A/B-*RNAi plants (Fig. 4B–E). Thus, specific defense responses are activated in *StSERK3A/B-*RNAi plants in response to Pep-13, despite highly reduced ROS formation and MAP kinase activation.

### Morphological and metabolic alterations of *StSERK3A/B-*RNAi plants

**Figure 5:**
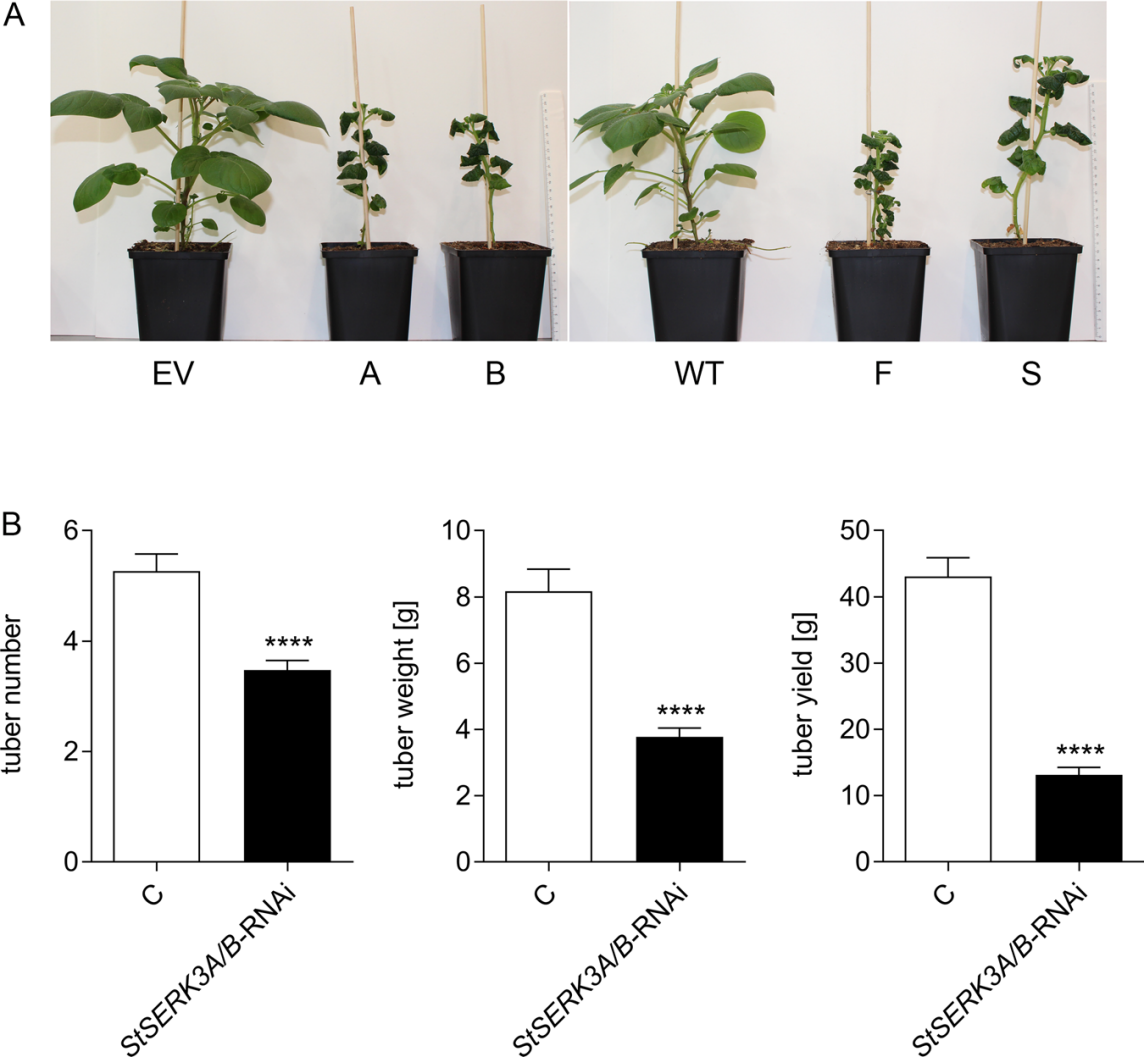
Phenotype and tuber yield in *StSERK3A/B-*RNAi plants. (**A**) Phenotype of *StSERK3A/B-*RNAi plants (A,B,F,S) compared to wild type (WT) and empty vector (EV) control plants grown in phytochambers. Scale bars represent  30 cm. (**B**) Tuber number per plant (**C**: n = 44, *StSERK3A/B-*RNAi: n = 88), average weight (C: n = 232, *StSERK3A/B-*RNAi: n = 306) and yield per plant (**C**: n = 44, *StSERK3A/B-*RNAi: n = 88) from phytochamber grown control (wild type and empty vector plants) and *StSERK3A/B-*RNAi plants. Data were obtained in three experiments. Statistical analyses were performed using two-tailed Mann-Whitney U test (****p < 0.0001).

*StSERK3A/B-*RNAi plants displayed major alterations in their phenotype (Fig. 5A). These included dwarfism in tissue culture, darker green leaves with a crinkled surface and leaf curling, resulting in a reduced expansion of the leaves. A delay in senescence was accompanied by reduced numbers and weight of tubers compared to control plants, leading to decreased overall tuber yield (Fig. 5B). The striking phenotype of the *StSERK3A/B-*RNAi lines is reminiscent of a brassinosteroid-deficiency phenotype observed in other plants^18,19^.

**Figure 6:**
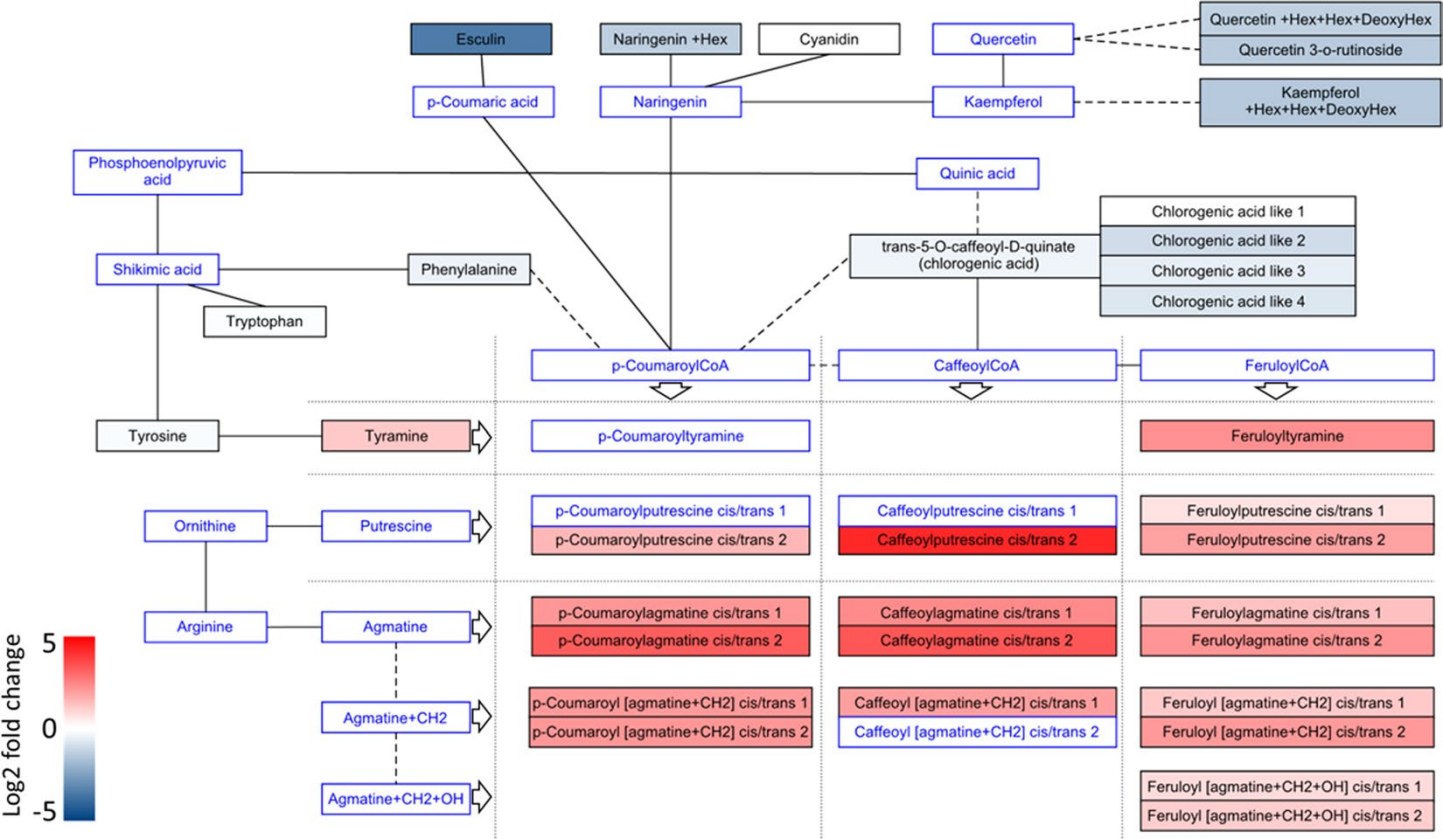
Changes in secondary metabolite levels in untreated potato leaves upon *StSERK3A/B* RNA interference. The relative abundances of metabolites from LC-MS analyses are mapped as log2 fold changes of *StSERK3A/B*-RNAi vs control (wildtype and empty vector) on metabolic pathways. Data are derived from three independent experiments (n = 41 for control plants, n = 84 for *StSERK3A/B-*RNAi plants).

**Figure 7:**
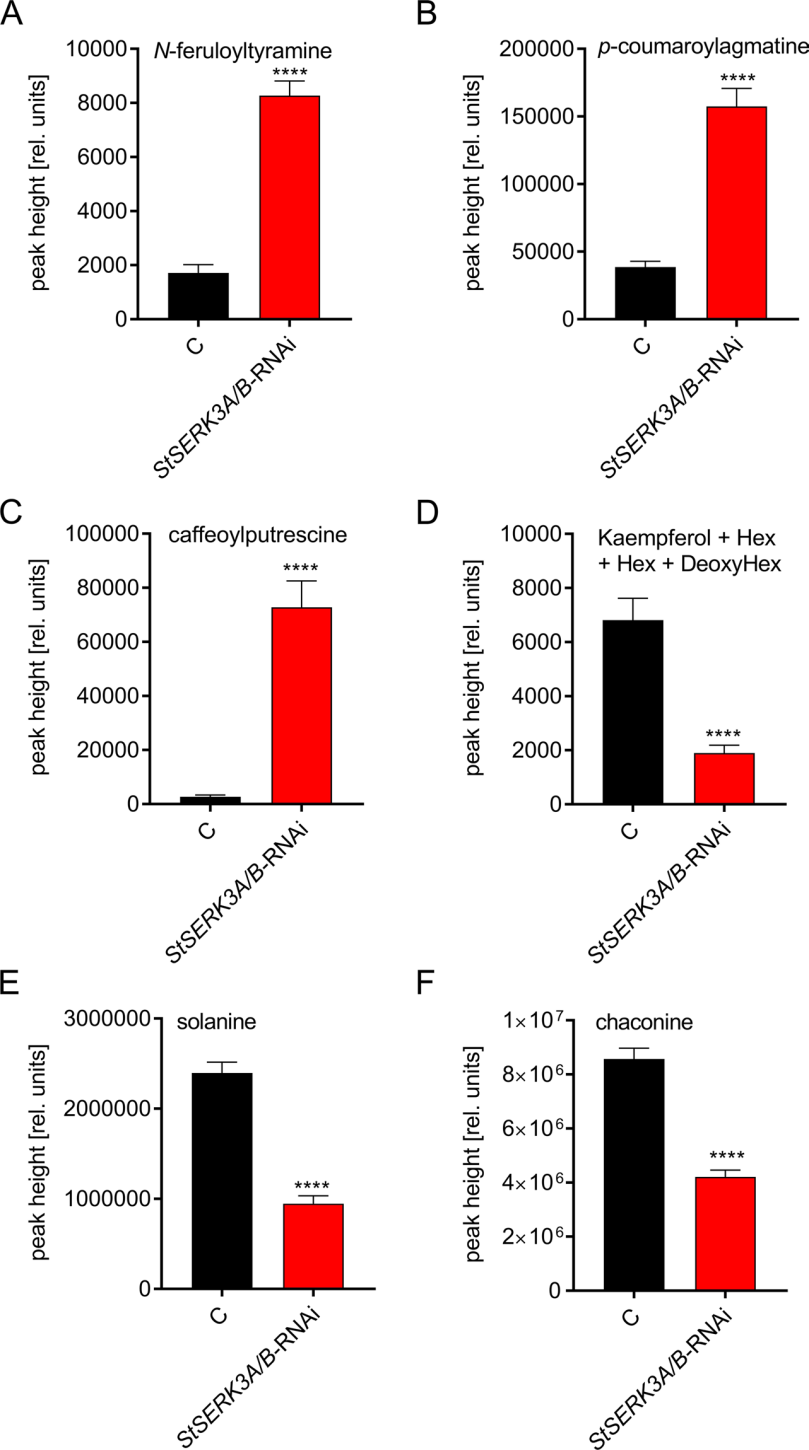
Differential accumulation of secondary metabolites in *StSERK3A/B-*RNAi plants. Relative quanitifcation of (**A**) *N*-feruloyltyramine; (**B**) *p*-coumaroylagmatine, (**C**) caffeoylputrescine, (**D**) kaempferolglycoside, (**E**) solanine, (**F**) chaconine by their ion response in LC-MS analyses. Data are derived from three independent experiments (n = 41 for control plants, n = 84 for *StSERK3A/B-*RNAi plants) Statistical evaluation was performed using Mann-Whitney two-tailed U test. ****P < 0.0001.

To further characterize differences between wild type and *StSERK3A/B-*RNAi plants, metabolites of methanolic extracts from untreated leaves of phytochamber-grown control and *StSERK3A/B-*RNAi plants were analysed using UPLC-ESI-QTOF-MS and -MS/MS in three independent experiments. Metabolites were identified based on analytical standards, by MS/MS similarity, data base search or MS/MS interpretation (Supplementary Table S1 and Supplementary Figs. S3–S6). Two experiments with higher replicate numbers, performed in 2017 and 2018 with 8 to 10 replicates per line, demonstrated many more significant common changes, whereas the first experiment of 2016 with only two to four biological replicates could not support all results due to the lack of statistical power (Supplementary Table S1). Despite this discrepancy, all three experiments were evaluated for the data shown in Figs. 6 and 7.

With more than 2000 metabolite features detected, we observed changes in branches of the phenylpropanoid pathway. Hydroxycinnamic acid amides, typical defense compounds of potato, were present at enhanced levels in *StSERK3A/B*-RNAi lines (Supplementary Table S1, Fig. 6). The fold changes varied from only minor increases up to factors of more than 5 fold, as visualized with bar charts for *N*-feruloyltyramine, p-coumaroylagmatine and caffeoylputrescine (Fig. 7A–C). The biogenic amines, putrescine and agmatine, were not detected in our experiments, whereas the levels of tyramine, a precursor of *N*-feruloyltyramine, were significantly enhanced.

In contrast to the enhanced levels of specific hydroxycinnamic acid amides, those of a number of coumarin and flavonoid compounds were significantly reduced (Fig. 6, Supplementary Table S1). The highest reduction was observed for esculin, a glycoside of the coumarin esculetin, with 10 fold lower levels in *StSERK3A/B*-RNAi plants. Similarly, flavonoids such as kaempferol and quercetin derivates displayed significantly reduced abundance (Figs. 6, 7D, Supplementary Table S1). Another class of compounds with reduced abundance in *StSERK3A/B*-RNAi lines was identified as chlorogenic acid derivatives. Four peaks with identical MS/MS were detected (Supplementary Fig. S3) with reduced abundance in the RNAi lines, suggesting that these chlorogenic acid-like compounds are derived from the same pathway. Finally, the steroidal glycoalkaloids solanine and chaconine, identified by MS-MS and analytical standards, were both significantly lower in *StSERK3A/B*-RNAi plants (Fig. 7E,F; Supplementary Table S1). In summary, elevated levels of hydroxycinnamic acid amides correlated with a concomitant reduction in the levels of coumarin and flavonoid compounds, suggesting that the common precursor of these pathways, coumaroyl-CoA, is preferentially converted by the HCAA branch of the phenylpropanoid pathway in *StSERK3A/B*-RNAi lines.

## Discussion

Reduced ROS formation and loss of MAP kinase activation in *StSERK3A/B*-RNAi lines suggests a requirement of StSERK3A/B for perception of Pep-13 in potato. ROS formation is a hallmark of early defense responses to pathogen and PAMP treatment^20^. In Arabidopsis, perception of PAMPs by a receptor complex comprising the PRR and BAK1 has been shown to activate the cytoplasmic RLK BOTRYTIS INDUCED KINASE 1 (BIK1), which subsequently phosphorylates and activates the ROS-forming enzyme RBOHD^21,22^. In Arabidopsis *bak1* mutants, the oxidative burst and MAP kinase activation in response to treatment with the PAMPs flg22 or elf18 are significantly reduced^16,17,23,24^, highlighting the importance of AtBAK1 for PAMP responsiveness. However, the degree of reduction varies in different *bak*1 mutants^17^. Moreover, in response to bacterial infection, Arabidopsis *bak1-4* mutants still show a reduced oxidative burst, suggesting redundancy^25^. Indeed, a double mutant defective in BAK1 (*bak1-5*) and the gene encoding the LRR-RLK SERK4/BKK1 shows even higher reduction in ROS formation and MAPK activation than *bak1-5* alone^23^. Searches for SERK4 homologous sequences from potato revealed highest sequence homology to PGSC0003DMP400047882 (Sotub04g027320), which is annotated as SERK1 in the potato genome database (http://solanaceae.plantbiology.msu.edu). Since transcript levels from this gene are not affected by the RNAi construct (Fig. 2D), we conclude that StSERK3A and B are required for Pep-13-induced ROS formation and MAP kinase activation in potato.

Despite the inability of *StSERK3A/B-*RNAi plants to accumulate ROS in response to Pep-13 (Fig. 3A,B) and to activate MAP kinases (Fig. 3E), they show defense gene activation upon treatment with Pep-13 (Fig. 4), which is similar to or even higher than that in wild type plants. Thus, the early responses that occur within minutes, i.e. ROS formation and MAPK activation, are clearly different from the later responses that are detectable after hours, i. e. defense gene activation.

This is in contrast to reports from other plants in which reduced *BAK1* expression also affects late responses, such as PAMP-induced cell death or growth inhibition. For example, the cell death response to the *Phytophthora* elicitin INF1 was reduced in *Nicotiana benthamiana* plants that were transiently silenced for *NbBAK1* expression^8^. Also, Arabidopsis *bak1* mutants displayed reduced growth inhibition in response to flg22^17^. On the other hand, in accordance with our data, potato plants silenced for BAK1 with a *StSERK3A*-specific RNAi construct showed Pep-13-inducible expression of three defense genes^26^, which led the authors to conclude that Pep-13 induces immunity in a SERK3/BAK1-independent manner. Our data do not support this conclusion, since Pep-13 neither induces ROS formation, nor activates MAPK in *StSERK3A/B*-depleted plants. Thus, our data show that perception of Pep-13 is dependent on StSERK3A/B.

The activation of defense responses in a BAK1-depleted background has been reported before^27^. In Arabidopsis *bak1* mutant plants, defense gene activation and cell death is elicited by treatment with endogenous plant peptide signals, such as Pep2^28^, which act as damage-associated molecular patterns (DAMPs). Apparently, Arabidopsis can sense the absence of BAK1 and responds with the activation of immune responses^29^. Thus, similar to elicitation by Pep2 in Arabidopsis *bak1* mutants, Pep-13 treatment of StSERK3A/B-depleted potato plants results in the activation of immune signaling.

The morphological alterations that were observed in all *StSERK3A/B*-RNAi plants might be a consequence of enhanced activation of immune responses, i.e. autoimmunity. In general, autoimmunity is accompanied by reduced growth, enhanced levels of salicylic acid, constitutive expression of defense genes as well as spontaneous lesion formation^30^. In potato, such a phenotype was observed in plants with reduced expression of *StSYR1*, a syntaxin required for the formation of callose-containing papillae^31^. In contrast, the *StSERK3A/B*-RNAi plants described here did not display spontaneous lesions (Fig. 5), nor constitutive defense gene expression (Fig. 4). Rather, the phenotype of *StSERK3A/B*-RNAi plants is more reminiscent of brassinosteroid-deficiency, with darker green curled leaves, decreased development and, most strikingly, delayed senescence (Fig. 5). Similarly, silencing of BAK1 in *Nicotiana benthamiana* leads to a morphological phenotype of crinkled leaves and dwarf stature^32^. In analogy to these reports, we would expect that brassinosteroid perception is impaired in the *StSERK3A/B*-RNAi plants^33,34^.

Along with the striking phenotype of the *StSERK3A/B*-RNAi plants, major alterations in the metabolite pattern of untreated transgenic plants compared to control plants were observed. The central precursor for the different branches of the phenylpropanoid pathway, 4-coumaroyl-CoA, was differentially channeled into the formation of HCAAs, while flavonoid levels were reduced (Fig. 6). As typical phytoalexins of *Solanaceous* plants, levels of HCAAs were at least threefold higher in the transgenic lines. Caffeoylputrescine, a compound which is found in a number of Colorado Potato Beetle-resistant wild species of *Solanum*^35^, is more than 25 times more abundant in *StSERK3A/B-*RNAi lines than in control lines. These observations correlate with reports that Arabidopsis brassinosteroid-deficient mutants contain higher amounts of aliphatic and indolic glucosinolates, typical defense compounds of Arabidopsis^36^. Moreover, exogenous application of brassinosteroids to Arabidopsis seedlings reduced the levels of glucosinolates, suggesting that brassinosteroids negatively affect defense compounds^36^. Our observation that *StSERK3A/B-*RNAi plants contain higher levels of defense metabolites is also in accordance with the analysis of Arabidopsis *serk1-3serk3-2* roots, which had higher levels of aliphatic glucosinolates as well as 4-methoxy-3-indol-3-ylmethyl glucosinolate^37^. The latter compound is a substrate of the atypical myrosinase PEN2^38,39^, which is required for penetration resistance against nonhost pathogens, such as *P. infestans*^40^.

In conclusion, silencing of *StSERK3A/B* in stably transformed potato plants not only altered early responses to the PAMP Pep-13, but resulted in major changes in metabolic pathways, emphasizing the central importance of StSERK3A/B for both developmental and stress-adaptation responses. The ability of *StSERK3A/B*-depleted plants to activate defense upon Pep-13 treatment, despite impaired early PAMP responses, suggests that potato can sense and compensate the loss of the PAMP co-receptor StSERK3A/B.

## Methods

### Cultivation of potato plants

Potato plants (*Solanum tuberosum* cv. Désirée) were cultivated in sterile tissue culture in a phytochamber (16 h light, ~140 µE, 22 °C). Plants were transferred to steam-sterilized soil and grown for four weeks under long day conditions in a phytochamber (16 h light, ~140 µE, 60% humidity, 20 °C).

### Generation of *StSERK3A/B-*RNAi transgenic potato plants

To generate the *StSERK3A/B* RNAi construct, the primers 5′-TGATGATGTCATGTTGCTAGATTG-3′ and 5′-CGGGTCGTAGATTATAAGTGGAGT-3′ were designed from a potato EST (MICRO.11825.C1) and used for amplification of a 320 bp fragment from potato genomic DNA. The RNAi fragment was transferred to pENTR/D-TOPO (Invitrogen) and cloned via Gateway LR cloning (Invitrogen) into pHELLSGATE8^41^. Potato plants (*Solanum tuberosum* cv. Désirée) were transformed with *Agrobacterium tumefaciens* AGL-0^42^ carrying the *StSERK3A/B-*RNAi binary vector. Generation of transgenic plants was performed as previously described^43^.

### Leaf disk and infiltration assay

Leaf disks were cut out from 4-week-old potato plants with a biopsy puncher (4 mm diameter) and placed with the abaxial side onto the surface of 250 µl of water in a 96-well plate. The plate was incubated overnight at 22 °C in the dark. Water from the wells was removed and 100 µl sterilized fresh water per well was added. The plate was incubated for 30 min in the phytochamber (20 °C). Elicitation was performed by adding 5 nM Pep-13 or the nearly inactive analog W2A^11^. For whole plant assays, PAMP treatment was performed by infiltrating a 100 µM elicitor solution into the abaxial side of leaves of 3-week-old potato plants growing in a phytochamber.

### RNA expression analyses

RNA was isolated from potato leaves or leaf disks as described^12^. DNase digestion (RNase-free DNase Set, Qiagen) and cDNA synthesis using Maxima H Minus First Strand cDNA Synthesis Kit (Thermo Fisher Scientific) were performed according to the manufacturer’s instructions. For quantitative PCR, Maxima Probe qPCR MasterMix (Thermo Fischer Scientific) was used and the samples were run on an Mx3005P qPCR system (Agilent).

The following primers and real time probes were used: for *StSERK3A/B*: 5′-TGTTTGGCTACGGAGTTATGC-3′, 5′-GCAAGTCGAGCAAGATCAAA-3′ and Roche Universal Probe Library Probe #61; for *StFAD*: 5′-ATCATGCTATGGAGGCAACC-3′, 5′-TGGAGTTCCATCAAATTGGTAGT-3′ and Roche Universal Probe Library Probe #147; for *StTHT*: 5′-CCTCCTTAGAGGGCTTGCTT-3′, 5′-AGTACGGATGGCCCGTAGA-3′ and Roche Universal Probe Library Probe #144; for *St4CL*: 5′-TGCTGTTGTCCCAATGATAGA-3′, 5′-TGATCTAACAACAAAAGCCACTG-3′ and Roche Universal Probe Library Probe #7. The amplification of the endogenous control *StEF1α* was performed with 5′-CACTGCCCAGGTCATCATC-3′, 5′-GTCGAGCACTGGTGCATATC-3′ and Roche Universal Probe Library Probe #163. To differentiate *StSERK3A* and *StSERK3B*, the following primers were used: 5′-CGTGAACTACAAGTTGCGTCG-3′ and 5′- CCATCAGCTAACCGGCCTTTA-3′, as well as 5′-CCGATACTTTTAACCACAGTCACTT-3′ and 5′-GAAGCTGGAGGAGTATCCAATG-3′, respectively. *StSERK1* transcripts were amplified using the primers 5′-TTACAACTGCTGTGCGTGGT-3′and 5′-TCTGAAGACTTCCCTGTGGAC-3′. Data were analyzed using Microsoft Excel 2013 and GraphPad Prism 7.04 (www.graphpad.com).

### ROS assay

ROS analyses were performed as described^44^ with the following modifications: Each well contained 200 µl of water supplied with 5 µM luminol L-012 (Wako Chemicals), 2 µg horseradish peroxidase (Fluka) and 5 nM Pep-13 or W2A peptide.

### Immunoblot analysis

Protein extraction was performed as described^45^.

### Liquid chromatography-mass spectrometry measurements

Leaf disks from 4-week-old potato plants were cut out and methanolic extracts were prepared as described^46^.

Chromatographic separations were performed as previously described^47^ with the following modifications: The binary gradient was applied at a flow rate of 150 μL/min: 0 to 1 min isocratic 95% A (water/ formic acid, 99.9/0.1 [v/v]) and 5% B (acetonitrile/formic acid, 99.9/0.1 [v/v]); 1 to 10 min linear from 5 to 60% B; 10 to 10.2 min linear to 95% B; 10.2 to 12 min isocratic 95% B; 12 to 14 min isocratic 5% B. Eluting compounds were detected from m/z 50 to 1000 using a micrOTOF- Q II hybrid quadrupole time-of-flight mass spectrometer (Bruker Daltonics) equipped with an Apollo II electrospray ion source in positive ion mode using the following instrument settings: nebulizer gas: nitrogen, 1.4 bar; dry gas: nitrogen, 6 L min^−1^, 190 °C; capillary: 5000 V; end plate offset: −500 V; funnel 1 RF: 200 V; funnel 2 RF: 300 V; in-source CID energy: 0 V; hexapole RF: 100 V; quadrupole ion energy: 3 eV; collision gas: nitrogen; collision energy: 5 eV; collision RF: 300 Vpp; transfer time: 70 μs; prepulse storage: 5 μs; pulser frequency: 10 kHz; spectra rate, 3 Hz. Mass spectra were acquired in centroid mode. Mass calibration of individual raw data files was performed on lithium formate cluster ions obtained by automatic infusion of 20 mL 10 mM lithium formate in isopropanol/water/formic acid, 49.9/49.9/0.2 (v/v/v) at a gradient time of 12 min using a diverter valve.

To reveal a comprehensive MS/MS library for structural annotations of compounds, the autoMS/MS method of the Bruker Otof control software was optimized. Ions were selected for MS/MS according to their intensity (highest first) and an intensity threshold of at least 500 counts, isolation width 0.5 Da, active exclusion after 2 spectra, reconsideration of excluded ions after 5 spectra. Preferred charge was 1+ or 2+, single charged ions were fragmented with 15 eV collision energy, double charge ions with 25 eV. For selected metabolites, the MS/MS collision energy was modified for optimized fragmentation.

Metabolite profiling was performed in MetaboScape 3.1 (Bruker Daltonics). Data files were assigned to sample groups of *StSERK3A/B-*RNAi lines “ABSF” and controls “empty vector and WT” with distinction of biological sampling in year 2016, 2017, 2018. The following settings were applied: Peak picking from 0.6 min to 10.5 min with intensity threshold: 1000 counts; minimum peak length: 9 spectra; re-extract feature if detected in 21 of 139 analyses; consider feature if found in 40 of 139 analyses or in 75% of samples in one sample group; mass calibration from 12.05 min to 12.2 min on lithium formate. [M + H]^+^ was set as primary ion, [M + Na]^+^ annotated as an adduct if the EIC correlation was above R = 0.9. Peak area was selected as an indicator for feature abundance. AutoMSMS data were mapped on the intensity matrix with the following settings: m/z tolerance 100 ppm, retention time (RT) tolerance 6 seconds.

Quantification of alpha-solanine (m/z 868.505, RT 5.8) and alpha-chaconine (m/z 852.510, RT 6.0) was performed on extracted ion chromatograms in QuantAnalysis 4.4 (Bruker), because peaks were not merged and quantified correctly by automated processing due to their width and peak shape.

Identification and annotation of the compounds was based on comparison of m/z and retention times to analytical reference standards, comparison of MS/MS patterns to analytical standards, published former annotations of hydroxycinnamic acid amides^47^ and interpretation of tandem mass spectra in combination with retention time systematics (Supplementary Table S1, Supplementary Figs. S3–S6).

Metabolic pathways were created in PathVisio 3.0.0^48^ and the log2 fold change of *StSERK3A/B*-RNAi vs. controls (wildtype, empty vector) was mapped via color code using MetaboScape (Bruker). For pathway mapping, all data of the experiments 2016, 2017, 2018 were combined; single experiment fold changes and p-values (Student’s t-test) were calculated in Excel and presented as Supplementary Table S1. Data in Fig. 7 was processed using GraphPad Prism 7.04 (www.graphpad.com).

### Accession numbers

Sequence data from this article can be found in the *Potato Genomics Resource* database (Spud Database, Michigan, USA) under the following accession numbers:

*StSERK3A*: PGSC0003DMT400045607; *StSERK3B*: PGSC0003DMT400032797, *StFAD*: PGSC0003DMT400061220, *St4CL*: PGSC0003DMT400036886, *StTHT*: PGSC0003DMT400038291.

## Supplementary Information

Supplementary Information
