# Supplementary material for: Early Pep-13-induced immune responses are SERK3A/B-dependent in potato
Source: Sci Rep. 2019 Dec 5;9:18380. doi: 10.1038/s41598-019-54944-y (PMC6895089; doi:10.1038/s41598-019-54944-y)
Supplement: Supplementary file 1 — Supplementary Information [file 41598_2019_54944_MOESM1_ESM.pdf]

Early Pep-13-induced immune responses are SERK3A/B-dependent in potato

Linda Nietzschmann, Karin Gorzolka, Ulrike Smolka, Andreas Matern, Lennart Eschen-Lippold, Dierk Scheel, Sabine Rosahl*

Department Biochemistry of Plant Interactions, Leibniz Institute of Plant Biochemistry, Weinberg 3, D-06120 Halle (Saale), Germany

A

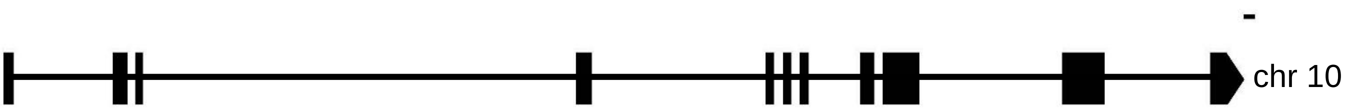

B

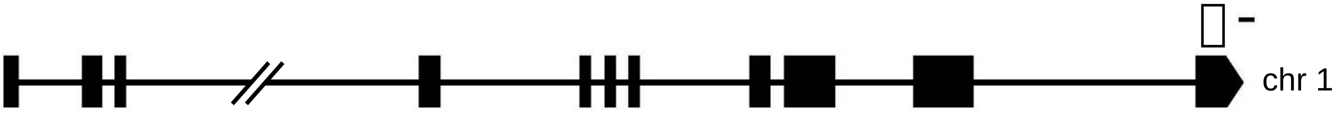

C

StSERK3A

```
MDQSVLAIWVFLCLIGLLLNLSMVAGNAEGDALNALKTNLADPNSVLQSWDATLVNPCTW
FHVTCNNENSVTRVDLGNANLSGQLVPQLGQLQNLQYLELYSNNISGRIPNELGNLTELV
SLDLYLNNLNGPIPPSLGKLQKLRFLRLNNNSLNEGIPVSLTTIVALQVLDLSNNHLTGP
VPVNGSFSLFTPISFANNQLEVPPVSPPPPLPPTPSSSSSVGNSATGAIAGGVAAGAALL
FAAPAIFIAWWRRRKPQDHFFDVPAEEDPEVHLGQLKRFSLRELQVASDNFSNRNILGRG
GFGKVYKGRLADGSLVAVKRLKEERTQGGELQFQTEVEMISMAVHRNLLRLRGFCMTPTE
RVLVYPYMENGSVASRLRERPESEPPLDWPKRKRIALGSARGLAYLHDHCDPKIIHRDVK
AANILLDEEFEAVVGDFGLAKLMDYKDTHVTTAVRGTIGHIAPEYLSTGKSSEKTDVFGY
GVMLLELITGQRAFDLARLANDDDVMLLDWVKGLLKDKKYETLVDADLQGNYNEEEVEQL
IQVALLCTQSTPTERPKMSEVVRMLEGDGLAERWEEWQKEEMFRQDFNHVHHHTDWIIA
DSTSNIRPDELSGPR
```

D

StSERK3B

```
MMDQWVLGILGSASVFLCLIGLLLVPVYGNTEGDALNALKTTLADPNNVLQSWDPTLVNP
CTWFHVTCNSENSVTRVDLGNANLSGQLVPQLGQLSNLQYLELYSNNISGRIPYELGNLT
ELVSLDLYLNKLVGPIPDTLGKLQKLRFLRLNNNSLTGQIPILLTTVTSLQVLDLSNNNL
TGPIPVNGSFSLFTPISFANNPLDTPPASPPPPISPTPTSPGVGNSATGAIAGGVAAGAA
LLFAAPAILLAWWRRRKPEDHFFDVPAEEDPEVHLGQLKRFSLRELQVATDSFSNKNILG
RGGFGKVYKGRLADGTLVAVKRLKEERTQGGELQFQTEVEMISMAVHRNLLRLWGFCMTA
TERLLVYPYMANGSVASRLRERPESDPPLGWPIRKCIALGSARGLAYLHDHCDPKIIHRD
VKAANILLDEEYEAVVGDFGLAKLMDYKDTHVTTAVRGTIGHIAPEYLSTGKSSEKTDVF
GYGVMLLELITGQRAFDLARLANDDDVMLLDWVKGLLKDEKYETLVDADLQGNYNEEEVK
QLIQVALLCTQSSPMERPKMSEVVRMLEGDGLAERWEEWQKEEMFRQDFNHAHHPHTDWI
IADSTYNLRPDELSGPR
```

Supplementary Figure S1

E

```
StSERK3A -MDQSVLAIW----VFLCLIGLLLNLSMVAGNAEGDALNALKTNLADPNSVLQSWDATLV
StSERK3B MMDQWVLGILGSASVFLCLIGLLL--VPVYGNTEGDALNALKTTLADPNNVLQSWDPTLV

StSERK3A NPCTWFHVTCNNENSVTRVDLGNANLSGQLVPQLGQLQNLQYLELYSNNISGRIPNELGN
StSERK3B NPCTWFHVTCNSENSVTRVDLGNANLSGQLVPQLGQLSNLQYLELYSNNISGRIPYELGN

StSERK3A LTELVSLDLYLNNLNGPIPPSLGKLQKLRFLRLNNNSLNEGIPVSLTTIVALQVLDLSNN
StSERK3B LTELVSLDLYLNKLVGPIPDTLGKLQKLRFLRLNNNSLTGQIPILLTTVTSLQVLDLSNN

StSERK3A HLTGPVPVNGSFSLFTPISFANNQLEVPPVSPPPPLPPTPSSSSSVGNSATGAIAGGVAA
StSERK3B NLTGPIPVNGSFSLFTPISFANNPLDTPPASPPPPISPTPTSPG-VGNSATGAIAGGVAA

StSERK3A GAALLFAAPAIFIAWWRRRKPQDHFFDVPAEEDPEVHLGQLKRFSLRELQVASDNFSNRN
StSERK3B GAALLFAAPAILLAWWRRRKPEDHFFDVPAEEDPEVHLGQLKRFSLRELQVATDSFSNKN

StSERK3A ILGRGGFGKVYKGRLADGSLVAVKRLKEERTQGGELQFQTEVEMISMAVHRNLLRLRGFC
StSERK3B ILGRGGFGKVYKGRLADGTLVAVKRLKEERTQGGELQFQTEVEMISMAVHRNLLRLWGFC

StSERK3A MTPTERVLVYPYMENGSVASRLRERPESEPPLDWPKRKRIALGSARGLAYLHDHCDPKII
StSERK3B MTATERLLVYPYMANGSVASRLRERPESDPPLGWPIRKCIALGSARGLAYLHDHCDPKII

StSERK3A HRDVKAANILLDEEFEAVVGDFGLAKLMDYKDTHVTTAVRGTIGHIAPEYLSTGKSSEKT
StSERK3B HRDVKAANILLDEEYEAVVGDFGLAKLMDYKDTHVTTAVRGTIGHIAPEYLSTGKSSEKT

StSERK3A DVFGYGVMLLELITGQRAFDLARLANDDDVMLLDWVKGLLKDKKYETLVDADLQGNYNEE
StSERK3B DVFGYGVMLLELITGQRAFDLARLANDDDVMLLDWVKGLLKDEKYETLVDADLQGNYNEE

StSERK3A EVEQLIQVALLCTQSTPTERPKMSEVVRMLEGDGLAERWEEWQKEEMFRQDFNHVHHHHT
StSERK3B EVKQLIQVALLCTQSSPMERPKMSEVVRMLEGDGLAERWEEWQKEEMFRQDFNHAHHPHT

StSERK3A DWIIADSTSNIRPDELSGPR
StSERK3B DWIIADSTYNLRPDELSGPR
```

Supplementary Figure S1: Structure of *StSERK3A* and *StSERK3B* genes and proteins.
(A,B) Structure of the *StSERK3A* (A) and *StSERK3B* (B) gene. The position of the RNAi fragment in *StSERK3B* is shown as a white box. Scale bars represent 100 bp.
(C,D) Predicted structure of the *St*SERK3A and StSERK3B proteins. Predicted domains are indicated as follows: signal peptide (green), leucine zipper region (yellow), predicted leucine-rich repeats (blue), transmembrane domain (red) and the intracellular serine/threonine kinase domain (grey).
(E) Amino acid comparison of StSERK3A and StSERK3B. Different amino acids are labelled in yellow.

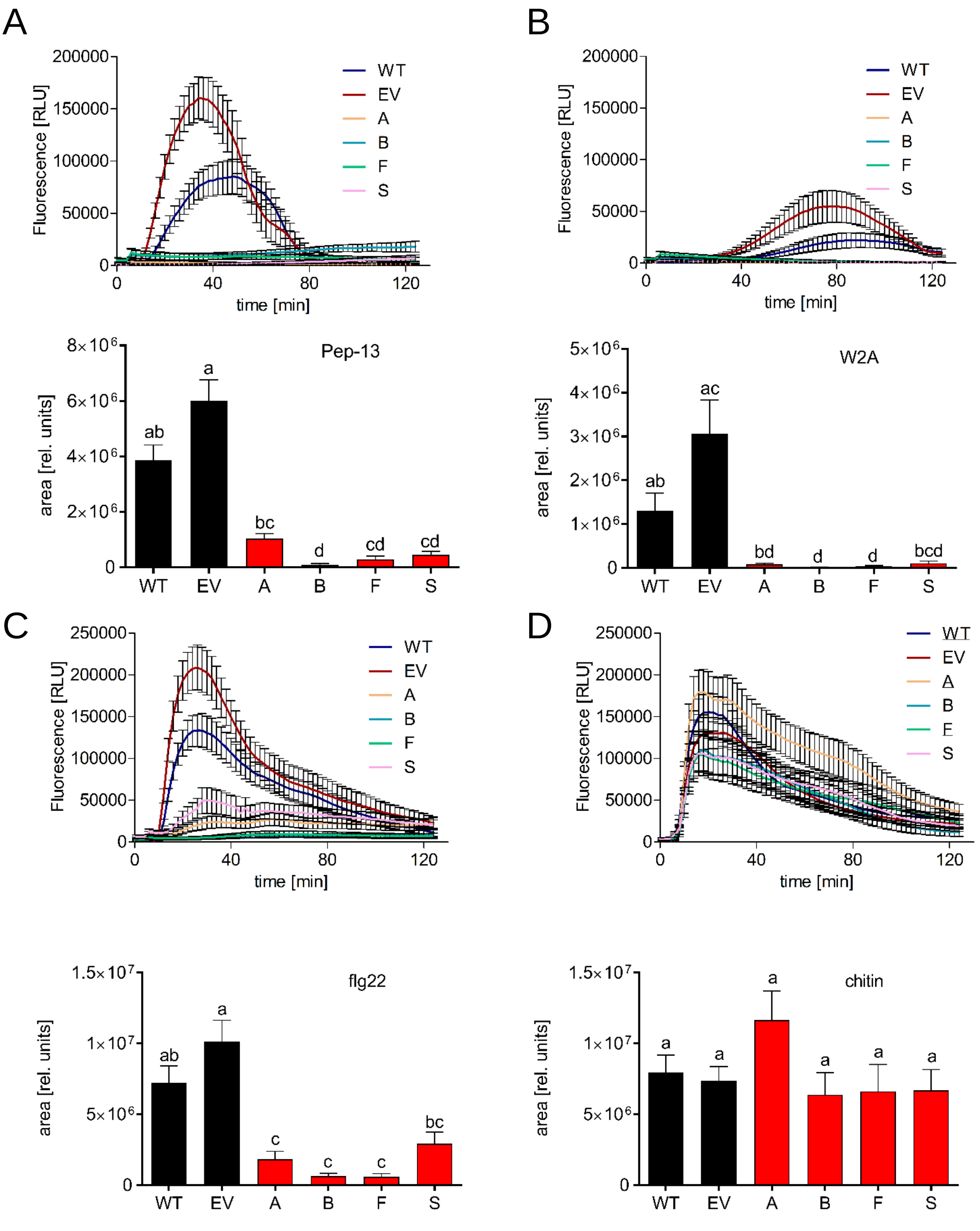

Supplementary Figure S2

E

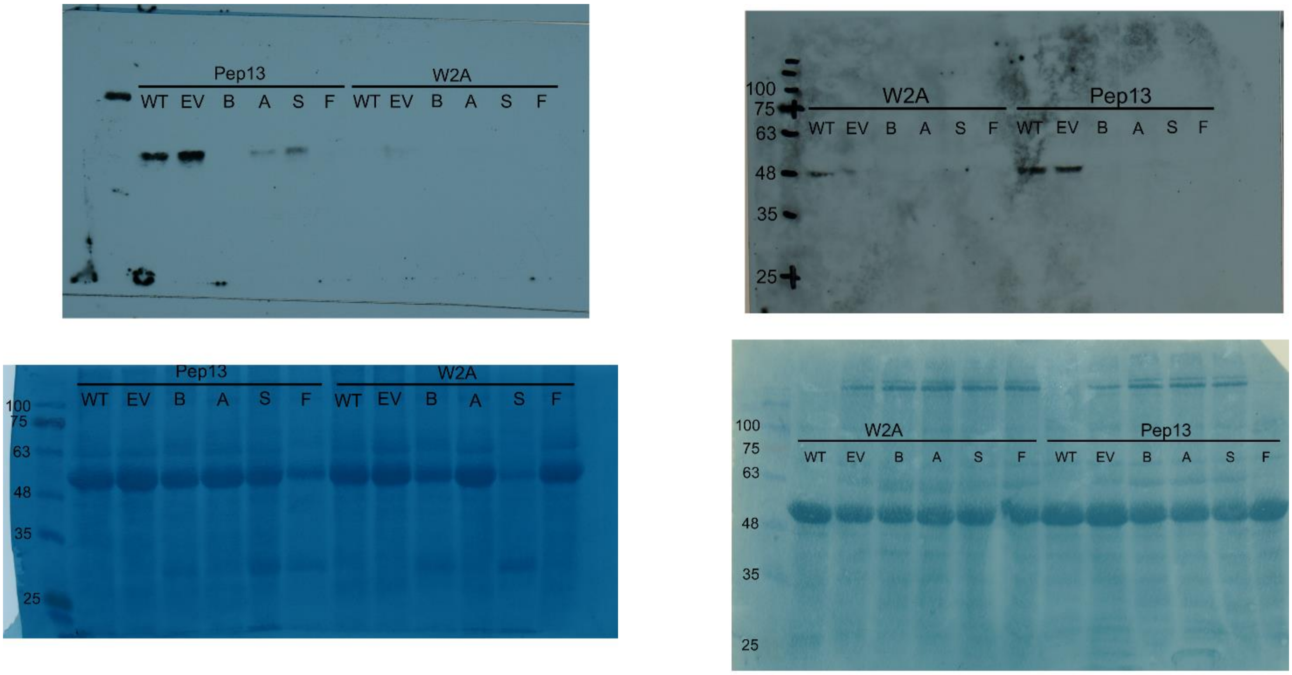

Supplementary Figure S2: ROS assays and MAP kinase activation in *StSERK3A/B*-RNAi plants. (A) - (D): Leaf disks from control (WT and EV) and *StSERK3A/B*-RNAi plants (A,B,F,S) were incubated in 5 nM Pep-13 (A), 5 nM W2A (B), 100 nM flg22 (C) or 100 µg/ml chitin (D) and assayed for luminol-based ROS production, as outlined in Figure 3. The upper panels show the original curves, the lower panels the calculated area under the curve. Data are derived from two independent experiments (n≥15). Statistical analyses were performed using two-way Anova. The summary of these data is shown in Fig. 3.

(E) Leaves from wild type, empty vector and StSERK3A/B-RNAi plants were infiltrated with Pep-13 or W2A and assayed for MAP kinase activation after 10 min. Protein extracts were subjected to Western blot analyses using anti-pTEpY antibodies (upper panels). The membrane was subsequently stained with amido black (lower panels). The original blots and membranes from two experiments are shown. The left part is also shown in Fig. 3E.

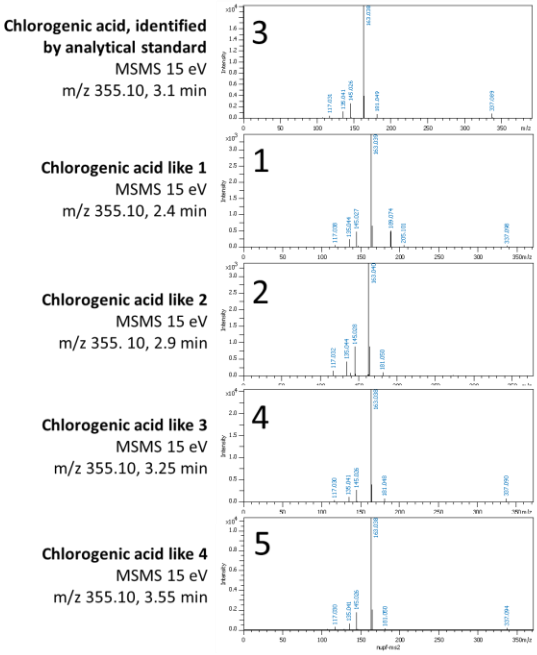

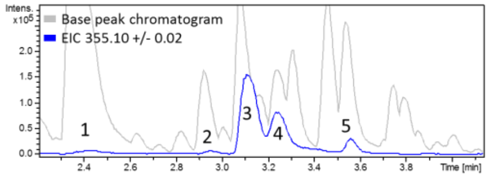

observed m/z	annotated sum formula	annotated structure
355.102 (precursor) 337.098: $-H_2O$	C16H18O9	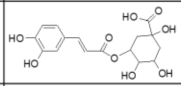
163.039	C9H6O3	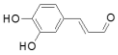
145.028	C9H4O2	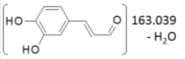
135.044	C8H8O2	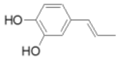
117.035	C8H6O	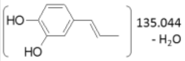

Supplementary Figure S3: MS/MS analysis of five consecutive peaks of m/z 355.10. Peak 3 was identified as chlorogenic acid (trans-5-O-caffeoyl-D-quinate) by retention time and MS/MS comparison with an analytical standard. The other four peaks exhibit nearly identical MS/MS and systematic retention time shifts.

The chromatogram shows the base peak and the extracted ion chromatogram of m/z 355.10 in an exemplary sample. Numbers indicate the peak with the respective MS/MS presented on the left. The table provides the interpretation of the MS/MS fragments with chlorogenic acid as the basic structure.

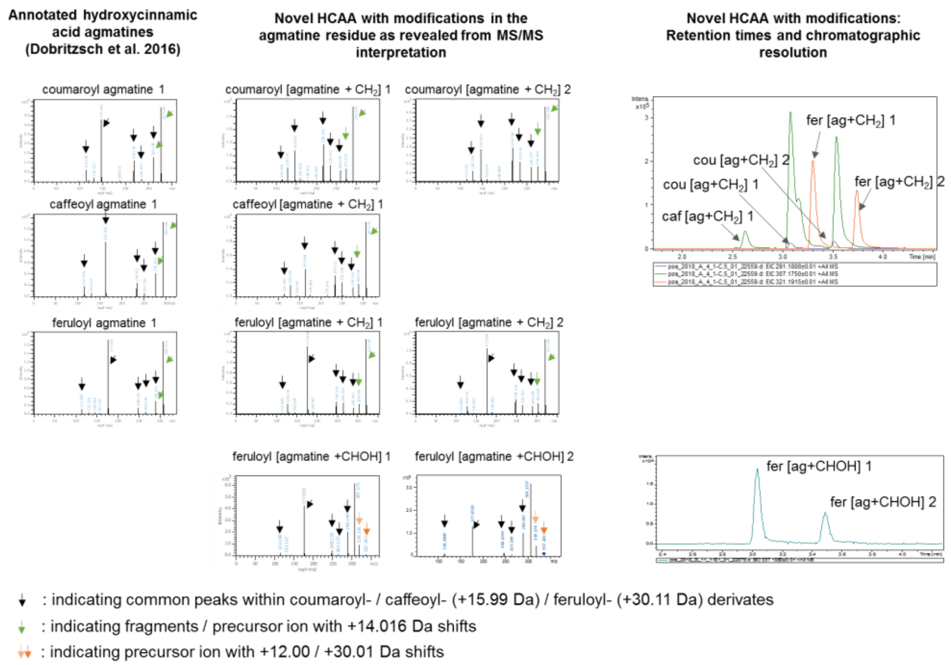

Supplementary Figure S4: MS/MS analysis of hydroxycinnamic acid amides. Seven compounds demonstrated high spectral similarity to hydroxycinnamic acid agmatine derivates. A postulated N-methylation of agmatine (+14.016 Da) explained all peaks in the MS/MS for all agmatine+CH_2 derivates. The interpretation of a -C-OH modification (+30.01 Da / +12.00 Da for C(-OH)) at the N of the agmatine for the “agmatine + CHOH” allowed the interpretation of all oberserved peaks. All compounds demonstrated the same chromatographic properties like the known hydroxycinnamic acid amides, e.g. two peaks due to cis / trans isomers.

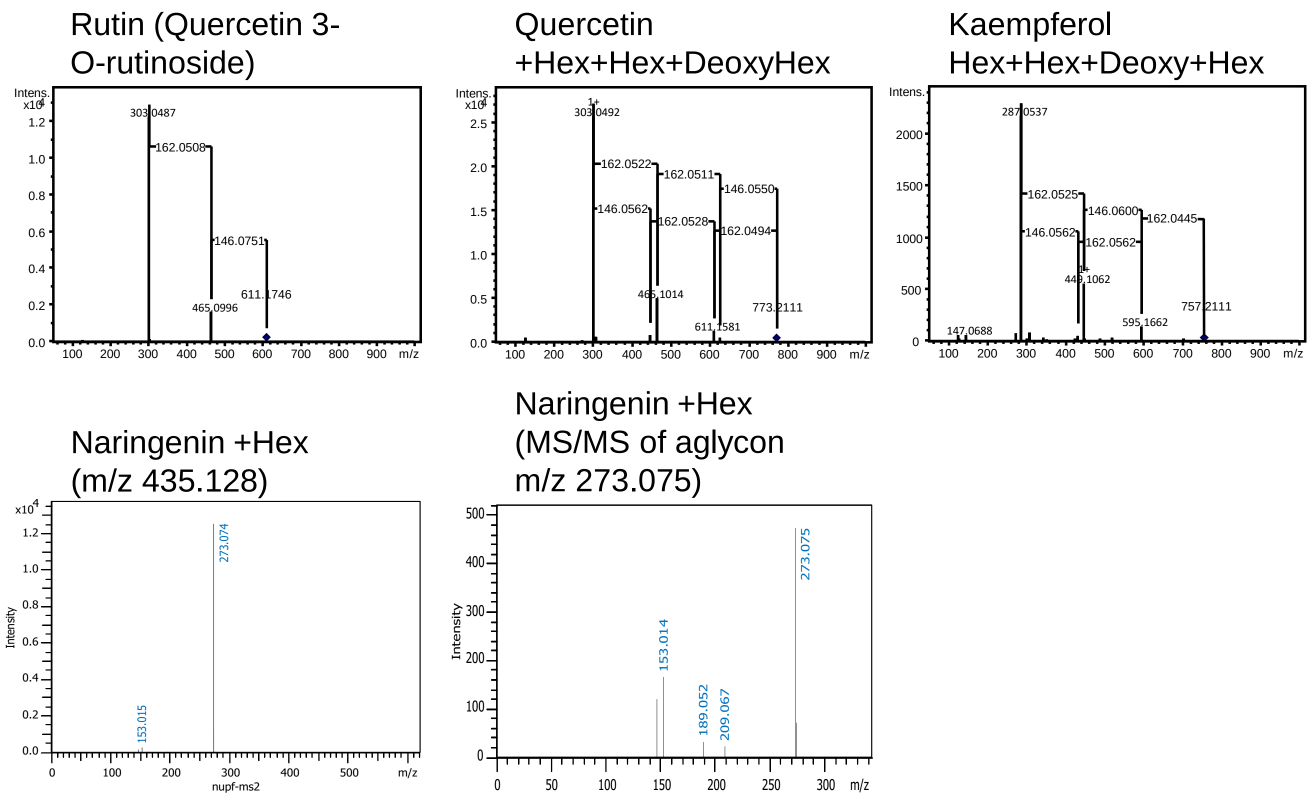

Supplementary Figure S5: MS/MS of glycosylated flavonoids in *Solanum tuberosum* extracts. Typical neutral losses of glycosylation were observed and interpreted as follows: 162.05: Hexose (Hex); 146.05: Deoxyhexose (DeoxyHex). m/z 303.049 is a common core structure observed in MS/MS of quercetins. m/z 287.053 is typical for kaempferol derivates (aglycon). However, other similar flavon structures (e.g. luteolin) of the same m/z cannot be excluded as aglycons. For Naringenin +Hex the MS/MS demonstrated the typical loss of 162.05. Additional MS/MS was performed on the resulting aglycon of m/z 273.075)

α-chaconine α-solanine

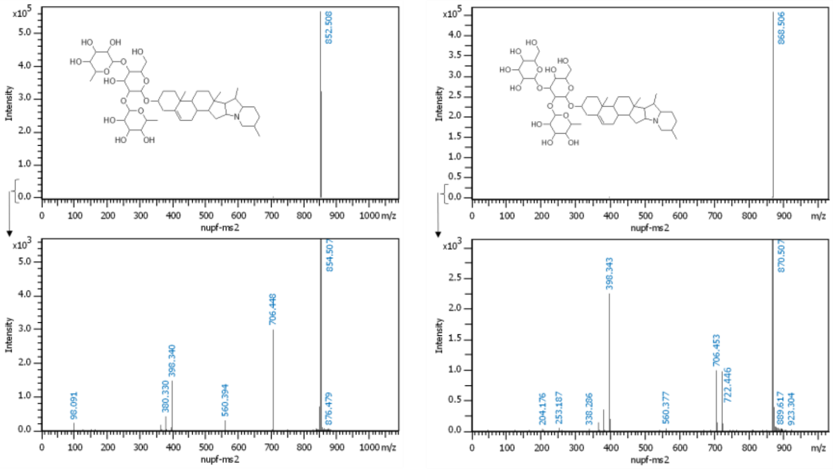

Interpretation of MS/MS:
m/z 398.343: aglycon
m/z 560.377: aglycon + Hex
m/z 706.453: aglycon + Hex + DeoxyHex
m/z 722.446: aglycon + Hex + Hex: solanine
m/z 852.508: aglycon + Hex + 2 DeoxyHex : chaconine
m/z 868.506: aglycon + 2 Hex + DeoxyHex: solanine

Supplementary Figure S6: MS/MS of alpha-chaconine and alpha-solanine from *Solanum tuberosum* extracts together with their molecular structure. Top: MS/MS, bottom: close up of the lower intensity ions that demonstrate the sequential loss of glycoside residues (hexose, deoxyhexose) and the common aglycon of m/z 398.34 (solanidine). Metabolite identification was validated by analytical standards.

Supplementary Table S1: Identified and annotated compounds in *StSERK3A/B*-RNAi lines with their log2-fold changes (*StSERK3A/B*-RNAi / Control) and p values as revealed by student's t-test and Mann-Whitney-U-Test in single experiments (2016, 2017, 2018) and for combined data.

	log2 Fold Change *StSERK3A/B*-RNAi / Control	Mann-Whitney U-test (p<0.01)	Student's T-Test p-value		log2 Fold Change *StSERK3A/B*-RNAi / Control			Mann-Whitney U-test (p<0.01)			p-value			Identification	Further information
	all experiments	all experiments	all experiments		2016	2017	2018	2016	2017	2018	2016	2017	2018		
L-Phenylalanine	**-0,44**	**< 0.0001**	**0,003**		-0,50	-0,08	-0,82	0,2005	0,61708	< 0.0001	0,232	0,680	0,000	analytical standard	
L-Tryptophan	**-0,20**	**0,01242**	**0,161**		-1,38	0,09	-0,26	0,0549	0,61708	0,00016	0,020	0,646	0,036	analytical standard	
L-Tyrosine	**-0,163**	**0,75656**	**0,443**		-0,59	0,69	-0,70	0,6101	0,85716	0,84148	0,341	0,020	0,076	analytical standard	
Tyramine	**1,12**	**< 0.0001**	**0,000**		0,82	0,28	2,88	0,1738	0,37346	< 0.0001	0,188	0,160	0,000	analytical standard	
N-feruloyltyramine	**2,25**	**< 0.0001**	**0,000**		2,75	2,01	2,32	0,0414	< 0.0001	< 0.0001	0,038	0,000	0,000	MS/MS interpretation	Dobritzsch et al. 2016
p-Coumaroylputrescine cis/trans 2	**1,55**	**< 0.0001**	**0,000**		-0,11	2,01	2,20	0,6171	< 0.0001	< 0.0001	0,544	0,000	0,000	MS/MS interpretation	Dobritzsch et al. 2016
Caffeoylputrescine cis-trans 2	**4,72**	**< 0.0001**	**0,000**		-0,35	7,54	2,78	1,0000	< 0.0001	< 0.0001	0,647	0,000	0,000	MS/MS interpretation	Dobritzsch et al. 2016
Feruloylputrescine 1	**0,23**	**0,22628**	**0,266**		0,10	0,97	0,00	1,0000	0,18684	0,65272	0,857	0,001	0,996	MS/MS interpretation	Dobritzsch et al. 2016
Feruloylputrescine 2	**1,89**	**< 0.0001**	**0,000**		0,28	3,96	1,35	0,7949	< 0.0001	< 0.0001	0,638	0,000	0,000	MS/MS interpretation	Dobritzsch et al. 2016
p-Coumaroylagmatine 1	**2,04**	**< 0.0001**	**0,000**		0,16	2,83	1,83	1,0000	< 0.0001	< 0.0001	0,656	0,000	0,000	analytical standard	Dobritzsch et al. 2016
p-Coumaroylagmatine 2	**3,23**	**< 0.0001**	**0,000**		0,86	3,99	3,66	0,0549	< 0.0001	< 0.0001	0,061	0,000	0,000	analytical standard	Dobritzsch et al. 2016
Caffeoylagmatine cis/trans 1	**2,34**	**< 0.0001**	**0,000**		-5,26	2,81	2,25	0,0105	< 0.0001	< 0.0001	0,010	0,000	0,000	MS/MS interpretation	Dobritzsch et al. 2016
Caffeoylagmatine cis/trans 2	**3,30**	**< 0.0001**	**0,000**		-2,69	3,59	3,19	0,2005	< 0.0001	< 0.0001	0,027	0,000	0,000	MS/MS interpretation	Dobritzsch et al. 2016
Feruloylagmatine cis/tran 1	**1,33**	**< 0.0001**	**0,000**		0,54	3,72	0,76	0,2501	< 0.0001	< 0.0001	0,199	0,000	0,000	analytical standard	Dobritzsch et al. 2016
Feruloylagmatine cis/trans 2	**2,20**	**< 0.0001**	**0,000**		0,95	3,53	1,52	0,0549	< 0.0001	< 0.0001	0,039	0,000	0,000	analytical standard	Dobritzsch et al. 2016
Coumaroyl [agmatine+CH2] cis/trans 1	**2,06**	**< 0.0001**	**0,000**		0,98	2,40	2,23	0,0151	< 0.0001	< 0.0001	0,006	0,000	0,000	MS/MS interpretation	Figure S4
Coumaroyl [agmatine+CH2] cis/trans 2	**2,07**	**< 0.0001**	**0,000**		1,51	2,13	2,75	0,0074	< 0.0001	< 0.0001	0,004	0,000	0,000	MS/MS interpretation	Figure S4
Caffeoyl [agmatine+CH2] cis/trans 1	**1,92**	**< 0.0001**	**0,000**		-0,95	1,42	2,07	0,6455	< 0.0001	< 0.0001	0,450	0,000	0,000	MS/MS interpretation	Figure S4
Feruloyl [agmatine+CH2] cis/trans 1	**1,14**	**< 0.0001**	**0,000**		0,82	1,61	0,94	0,0735	< 0.0001	< 0.0001	0,030	0,000	0,000	MS/MS interpretation	Figure S4
Feruloyl [agmatine+CH2] cis/trans 2	**2,07**	**< 0.0001**	**0,000**		1,57	2,55	1,84	0,0033	< 0.0001	< 0.0001	0,001	0,000	0,000	MS/MS interpretation	Figure S4
Feruloyl [agmatine+CH2OH] cis/trans 1	**0,73**	**0,93624**	**0,049**		1,25	1,02	0,62	0,2501	0,69654	< 0.0001	0,134	0,014	0,179	MS/MS interpretation	Figure S4
Feruloyl [agmatine+CH2OH] cis/trans 2	**1,02**	**0,13104**	**0,001**		1,49	0,65	1,36	0,4179	0,31732	< 0.0001	0,231	0,057	0,014	MS/MS interpretation	Figure S4
Esculin	**-3,50**	**< 0.0001**	**0,000**		-2,58	-3,58	-3,64	0,0969	< 0.0001	< 0.0001	0,039	0,000	0,000	analytical standard	
Naringenin + Hex	**-1,43**	**0,00034**	**0,002**		1,10	-2,47	-0,71	0,1260	< 0.0001	0,01828	0,033	0,017	0,182	MS/MS interpretation	Figure S5
Cyanidin	**0,02**	**0,85716**	**0,995**		0,63	-1,20	0,18	0,1585	0,00328	0,04884	0,158	0,034	0,065	analytical standard	
Quercetin+Hex+Hex+DeoxyHex	**-1,30**	**< 0.0001**	**0,000**		-0,81	-1,47	-1,10	0,6101	< 0.0001	< 0.0001	0,392	0,000	0,000	MS/MS interpretation	Figure S5
Quercetin 3-o-rutinoside (Rutin)	**-1,48**	**< 0.0001**	**0,000**		-0,28	-1,86	-1,47	0,5222	< 0.0001	< 0.0001	0,518	0,000	0,000	analytical standard	Figure S5
Kaempferol + Hex + Hex + DeoxyHex	**-1,50**	**< 0.0001**	**0,000**		-0,27	-1,76	-1,79	0,4413	< 0.0001	< 0.0001	0,548	0,000	0,000	MS/MS interpretation	Figure S5
trans-5-O-caffeoyl-D-quinate (chlorogenic acid)	**-0,39**	**0,00006**	**0,001**		-0,94	-0,50	-0,31	0,0969	< 0.0001	0,00008	0,053	0,000	0,000	analytical standard	Figure S3
Chlorogenic acid like 1	**0,02**	**0,90448**	**0,857**		-2,77	0,11	-0,34	0,0214	0,44726	0,4413	0,015	0,549	0,202	MS/MS interpretation	Figure S3
Chlorogenic acid like 2	**-0,97**	**< 0.0001**	**0,000**		-3,91	-1,04	-0,92	0,0151	< 0.0001	< 0.0001	0,010	0,000	0,000	MS/MS interpretation	Figure S3
Chlorogenic acid like 3	**-0,73**	**< 0.0001**	**0,002**		-1,92	-0,85	-0,57	0,0549	< 0.0001	0,00034	0,017	0,000	0,002	MS/MS interpretation	Figure S3
Chlorogenic acid like 4	**-0,54**	**0,00038**	**0,000**		-1,04	-0,67	-0,42	0,0969	< 0.0001	< 0.0001	0,048	0,000	0,000	MS/MS interpretation	Figure S3
Chaconine	**-1,02**	**< 0.0001**	**0,000**		-0,33	-1,33	-0,89	0,1031	< 0.0001	< 0.0001	0,059	0,000	0,000	analytical standard	Figure S6
Solanine	**-1,34**	**< 0.0001**	**0,000**		-0,28	-1,93	-1,19	0,1285	< 0.0001	< 0.0001	0,094	0,000	0,000	analytical standard	Figure S6

-5 0 5

Log2 fold change

p<0.01
